# From Genome to Phenotype: An Integrative Approach to Evaluate the Biodiversity of *Lactococcus lactis*

**DOI:** 10.3390/microorganisms5020027

**Published:** 2017-05-19

**Authors:** Valérie Laroute, Hélène Tormo, Christel Couderc, Muriel Mercier-Bonin, Pascal Le Bourgeois, Muriel Cocaign-Bousquet, Marie-Line Daveran-Mingot

**Affiliations:** 1 LISBP, Université de Toulouse, CNRS, INRA, INSA, Toulouse, France; valerie.laroute@insa-toulouse.fr (V.L.); pascal.lebourgeois@univ-tlse3.fr (P.L.B.); muriel.cocaign-bousquet@insa-toulouse.fr (M.C.-B.); 2 Département des Sciences Agronomiques et Agroalimentaire, équipe Agroalimentaire et Nutrition, Université de Toulouse, INP-Purpan, Toulouse, France; helene.tormo@purpan.fr (H.T.); christel.couderc@purpan.fr (C.C.); 3 Toxalim (Research Centre in Food Toxicology), Université de Toulouse, INRA, ENVT, INP-Purpan, UPS, Toulouse, France; muriel.mercier-bonin@inra.fr; 4 Université de Toulouse III, Université Paul Sabatier, F-31062 Toulouse, France

**Keywords:** *Lactococcus lactis*, diversity, genotype, phenotype, diacetyl, raffinose metabolism

## Abstract

*Lactococcus lactis* is one of the most extensively used lactic acid bacteria for the manufacture of dairy products. Exploring the biodiversity of *L. lactis* is extremely promising both to acquire new knowledge and for food and health-driven applications. *L. lactis* is divided into four subspecies: *lactis*, *cremoris*, *hordniae* and *tructae*, but only subsp. *lactis* and subsp. *cremoris* are of industrial interest. Due to its various biotopes, *Lactococcus* subsp. *lactis* is considered the most diverse. The diversity of *L. lactis* subsp. *lactis* has been assessed at genetic, genomic and phenotypic levels. Multi-Locus Sequence Type (MLST) analysis of strains from different origins revealed that the subsp. *lactis* can be classified in two groups: “domesticated” strains with low genetic diversity, and “environmental” strains that are the main contributors of the genetic diversity of the subsp. *lactis*. As expected, the phenotype investigation of *L. lactis* strains reported here revealed highly diverse carbohydrate metabolism, especially in plant- and gut-derived carbohydrates, diacetyl production and stress survival. The integration of genotypic and phenotypic studies could improve the relevance of screening culture collections for the selection of strains dedicated to specific functions and applications.

## 1. Introduction

Lactic acid bacteria (LAB) contain a variety of industrially important genera including *Enterococcus*, *Lactobacillus*, *Lactococcus*, *Leuconostoc*, *Oenococcus*, *Pediococcus*, and *Streptococcus*. Among them, the *Lactococcus* genus, belonging to the phylum *Firmicutes*, is closely related to the *Streptococcus* genus, both members of the *Streptococcaceae* family, and has 11 species: *L. lactis*, *L. raffinolactis*, *L. garviae*, *L. plantarum*, *L. piscium*, *L. chungengensis*, *L. fujiensis*, *L. taiwanensis*, *L. formosensis* and two newly identified species *L. hircilactis* and *L. laudensis* [1]. To date, *L. lactis* is the best known lactococcal species. It is one of the most frequently used microorganisms in the dairy industry and its use has the “generally recognized as safe” (GRAS) status. *L. lactis* is involved in the manufacture of various dairy products, both artisanal and industrial ones, such as (soft) cheese, buttermilk and sour cream. Its major role in dairy as dairy starter culture is to provide lactic acid at an efficient rate during milk fermentation. In addition to its role in the first acidification step, *L. lactis* contributes to the flavor of dairy products, notably due to its capacity to produce diacetyl and acetoin. *L. lactis* is also involved in microbial safety, with a high production of lactic acid but also of anti-microbial agents such as bacteriocins [2,3]. Until recently, *L. lactis* strains routinely used in food fermentation have been selected according to their technological properties (acidification rate, phages resistance…) and their capability to produce diacetyl, an aroma well-known for its buttery taste. However, the increasing demand for products with a wide range of new organoleptic properties has boosted investigations regarding the biodiversity of the species.

The aim of this review is to highlight the biodiversity of *L. lactis* by using an integrated approach. Three levels of diversity are explored: genetic, genomic and functional characteristics. Defining this diversity will enable rational selection of optimized candidates not only for dairy products but also for non-food applications, including white biotechnology or health issues [4].

## 2. Main Characteristics of the *L. lactis* Species

### 2.1. Taxonomic Features

*Lactococcus lactis* is a Gram-positive, non-sporulating, aerotolerant bacteria belonging to the *Streptococcaceae* family. The species is divided into four subspecies, *lactis*, *cremoris*, *hordniae* and *tructae* [5,6], and one Diacetylactis biovar. It is a part of mesophilic microorganisms involved in dairy fermented products, but only subsp. *lactis* and subsp. *cremoris* are of industrial interest. Before the development of molecular methods, phenoptypic features were classically used to discriminate the two subspecies. The ability to grow in 4% NaCl (m/v) at 40 °C and pH 9.2 and to degrade arginine were the main characteristics of *L. lactis* subsp. *lactis*, whereas *L. lactis* subsp. *cremoris* did not share these features.

In the early 1980s, genotyping methods replaced phenotypic characterization. The 16S rRNA sequence became the gold standard for species delineation, and differentiated the two subspecies with as little as 0.7% of nucleotide divergence. However, distinguishing between the two subspecies is of special importance in the elaboration of dairy products, particularly in cheese. Numerous molecular methods, including Amplified Ribosomal DNA Restriction Analysis (ARDRA) [7,8] and Southern blot hybridization of branched chain amino acid biosynthesis genes were then proposed [9]. Various subspecies-specific PCR have been developed. As a result, the mosaic structure of the histidine biosynthesis operon has been exploited. In contrast to *lactis* subspecies, a 200-bp insertion in the *hisZ* gene was reported in the *cremoris* subspecies, resulting in amplicons of different sizes [10,11]. Similarly, the polymorphism of the *gad* operon was used to design a subspecies-specific PCR [12]. This operon encodes glutamate decarboxylase (*gadB*) and its transporter (*gadC*) and is involved in the conversion of glutamate into γ-aminobutyric acid (GABA) [13]. The presence of deletions in the 3′ untranslated region of *gadB* was only observed for the *cremoris* subspecies. A PCR fragment spanning the 3′-UTR allowed for distinguishing between the two subspecies. Moreover, the presence or absence of an *Ase*I restriction site inside this amplicon has been correlated to the GAD+ or GAD− phenotype, respectively corresponding to Lactis and Cremoris phenotypes.

With the increasing amount of available data on bacterial genome sequences, the use of average nucleotide identity (ANI) is now a valuable tool for accurate species classification [14]. Cavanagh et al. [15] determined ANIb values (ANI calculated using the BLAST algorithm) of a set of 19 *L. lactis* genome sequences. In the same subspecies, ANIb values were comprised between 96.53% and 99.96%. In contrast, strains classified in different subspecies shared between 85.54% and 87.45% ANIb values. These values were below the threshold for species circumscription (<95%) [14] and could justify considering the *lactis* and *cremoris* subspecies as two different species.

Phenotypic and genotypic identifications are not always correlated. If the *lactis* genotype encompasses strains sharing the same phenotypic traits described above (termed the “Lactis” phenotype), the *cremoris* genotype is quite phenotypically heterogeneous. Although the two reference strains MG1363 and SK11 belong to the *cremoris* genotype, one displays the Lactis phenotype and the other the Cremoris phenotype [16,17]. This led to some confusion in the characterization of the two subspecies. Thus, genetic classification alone may not represent the phenotypic diversity, and both genotypic and phenotypic studies should be performed in tandem to accurately represent the multifaceted potential of a strain. However, our recent data based on Multi-Locus Sequence Typing (MLST) analysis of the *cremoris* genotype revealed two genetic lineages, one corresponding to strains with the Lactis phenotype and one with strains harboring the Cremoris phenotype.

### 2.2. Ecological Niches

*L. lactis* can colonize very different biotopes. The species is occasionally recovered as the subdominant population from traditional sourdoughs, brought to this complex ecosystem via the raw material [18,19]. Indeed, plant material is considered as the natural habitat of the subspecies *lactis*, where it usually occurs as an early colonizer, and is later replaced by species that are more tolerant to low pH values. Kelly et al. [20] found this bacterium in seeds prior to sprouting. The occurrence of bacteriocin producers probably favors the dominance of these strains. Among LAB, *Lactococcus* is one of main epiphytic and endophytic bacteria [21]. More specifically, *L. lactis* can inhabit different parts of plants including the stems of *Eucalyptus* [22], corn, peas [23], and the leaves of sugar cane [24]. The strains associated with plants rapidly grow and reach high cell densities in leaf tissue lysates due to their capacity to consume a broad range of carbohydrates and to their fewer amino acid auxotrophies [25]. Moreover, some strains could have positive effects on plant growth via their ability to solubilize or mineralize phosphate [26]. Although humans and animals are not a common host, they may contain *L. lactis* [6,27]. However, it is generally accepted that *L. lactis* originates from plant material. For example, the *L. lactis* subsp. *cremoris* Mast36 strain isolated from bovine mastitis possesses genes of plant origin and a cluster of genes associated with pathogenicity [28].

Raw milk is a well-known source of *L. lactis*, but it is plausible that milk is not its natural habitat but was rather colonized by the species after contact with dairy environment and plants. The animal’s environment appeared to play a role in the balance between the dominance of *L. lactis* and enterococci in goat milk [29]. The possible inoculation of the milk by *L. lactis* originating from hay was discussed. Milking machines were an important source of inoculation with microorganisms from milk including *L. lactis* [30,31]. Analysis of raw milk showed a reduction in the level of lactococci over time [32,33]. This was probably linked to sanitation practices and, particularly, the milking machines [30,31,34,35]. These results suggest that milking machines are a major reservoir of *L. lactis*.

Although the subspecies *lactis* has been found in various environments from plants to cattle and milk, in most cases, the strains displaying a *cremoris* genotype and a Cremoris phenotype (that can be regarded as the “true” subspecies *cremoris*) are only present in milk. Indeed, growth at 40 °C in 4% NaCl (m/v) at pH 9.2 and the capacity to utilize arginine are not required in milk, contrary to more stringent habitats. More detailed phylogenetic studies may be able to confirm the possible reductive evolution of this subspecies for its adaptation to milk. Due to its ability to colonize various biotopes, *Lactococcus lactis* subsp. *lactis* is generally considered to be genetically more diverse than the subspecies *cremoris*.

## 3. *Lactococcus lactis:* Multiple Levels of Diversity

### 3.1. Genetic Structure of L. lactis

The development of next generation sequencing (NGS) technologies has provided numerous complete or draft lactococcal genomes. The sequence of the *L. lactis* subsp. *lactis* IL1403 strain was released in 2001 [36]. Of the 83 *L. lactis* sequences available on public databases, 50 were published in 2015 and 2016 (Table 1). However, a robust phylogenetic analysis of the species from the core genome derived from these data has not yet been conducted. Up to now, the extent of genetic diversity has been explored by Multi Locus Sequence Typing (MLST) studies. MLST is a powerful technique based on the sequencing of a limited number of genes in the core genome [37]. It provides information on the population structure, the long-term epidemiology and the evolutionary history of the species. Indeed, the concatenated sequences of the set of genes represent one “signature” of the core genome and are used in phylogenetic analysis.

**Figure 1:**
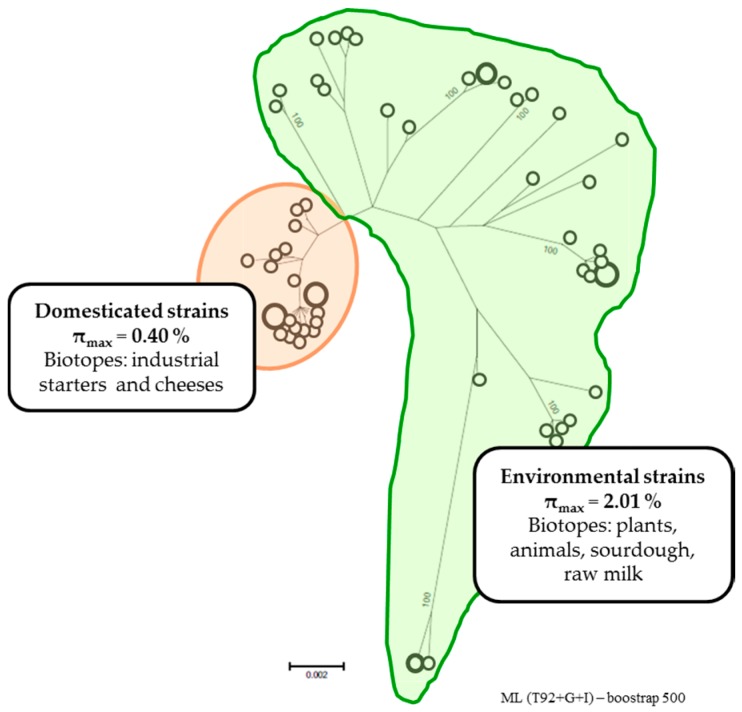
The two phylogenetic groups of the *Lactococcus lactis* subsp. *lactis*. The unrooted maximum likelihood tree (bootstrap 500, Tamura 3-parameter model) was constructed from the concatenated sequences of the six loci of MLST scheme from [41]. Open circles correspond to the different sequence type (ST). The size of the circles is proportional to the number of strains.

Several MLST studies were conducted with different sets of strains from both subspecies and from different origins. Rademaker et al. [38] used a subset of 89 strains of the two subspecies *lactis* and *cremoris* of dairy and non-dairy origin. Five genes were used for phylogenetic analysis. This revealed two major, distinct genomic lineages within the species. These lineages did not correlate with the phenotypic characterization of the two subspecies but did correlate with the genotypic identification. One genomic lineage consists of the *lactis* genotype and phenotype strains including biovar Diacetylactis. The second lineage encompasses isolates with the *cremoris* genotype but with the two Cremoris and Lactis phenotypes, underlining the fact that only identifying the genotype reflects the evolutionary history. However, this analysis was unable to differentiate the strains based on their origins. The same results were obtained by Fermandez et al. [39] using the same MLST scheme but a different set of strains (mainly isolated from traditional cheeses and raw milk). To increase the discriminatory potential of this method, Xu et al. [40] analyzed a partial sequence of 12 genes from 197 *L. lactis* strains isolated from natural homemade yogurt. As was the case in the previous studies, the two major lineages corresponding to the two subspecies were revealed. Despite the increased number of genes, the genetic distance between the two subspecies did not enable the distinction of clusters within the lineages. As suggested, these two subspecies could be considered as two different species, but the method lacks accuracy to distinguish the *L. lactis* species considered as a whole. A more precise phylogenetic study has been proposed for the *L. lactis* subsp. *lactis* [41]. The π_MAX,_ defined as maximum nucleotide diversity, was chosen as an indicator of the diversity within the subspecies because it is not directly sensitive to the size of the sample. A phylogenetic tree was constructed from the concatenated sequences of the six loci targeted in this new MLST scheme. Two of them, *glyA* and *recN*, belonged to the gene set identified as the most reliable predictor of whole genome relatedness [42]. The computed π_MAX_ for the subspecies lactis was 2.01%, a value within the range of values calculated for several species. The genetic structure clearly clustered the 36 *L. lactis* subsp. *lactis* strains of the study in two groups with different diversity level (Figure 1). The first group, with low genetic diversity, had a π_MAX_ of 0.4%, and clustered strains isolated from dairy starters or fermented products and involved in industrial milk processing. These strains could be considered as “domesticated”. The second group, with high diversity, had a π_MAX_ of 2.01%, and was identical to that of the whole subspecies. Clearly, it is the main contributor of the diversity of the subspecies with “environmental” strains isolated from various natural sources such as plants, animals and raw milk. According to the structure of the population described by this analysis, Passerini et al. [41] proposed classifying the subspecies *lactis* strains in “domesticated” versus “environmental” strains instead of dairy and non-dairy, as it is usually the case. The “domesticated” strains emerged more recently, probably due to the selective pressure of industrial processing. An alternative hypothesis would be that actual “domesticated” strains originate from very few strains isolated and used as commercial starters for standardized cheese production in the early 20th century. The “environmental” strains appeared first and their high genetic diversity explains their ubiquitous presence in various natural environments. The “environmental” status of strains isolated from raw milk reflects their plant origins.

### 3.2. Genomic Diversity

In addition to the allelic variation between genes conserved among strains (i.e., core genes), a second level of biodiversity related to the gene content shared by a few strains or specific to one strain (i.e., accessory genes) can be described. This accessory genome provides a given strain with wide adaptability and extending capacities such as the ability to colonize different ecological niches. The increasing amount of lactococcal genome sequences is undoubtedly a powerful tool to highlight these capacities. In the NCBI genome database (Table 1), the mean genome size of the two subspecies is quite similar (2504 kb ranging from 2245 kb to 2744 kb for the subspecies *lactis* and 2537 kb ranging from 2000 kb to 2862 kb for the subspecies *cremoris*). It should be noted that about a 30% difference between the smallest and the largest genomes has been found for the *cremoris* subspecies, indicating high fluctuation in strain-to-strain coding capacity. This difference is bigger than that found in the *lactis* subspecies (20%) and might reflect the large plasmid content in some strains of the *cremoris* subspecies. For a set of strains, the chromosome size has been obtained from the sequence data. The average chromosome sizes are 2491 kb and 2467 kb with about 10% to 15% difference between the extremes, for the subspecies *lactis* and *cremoris*, respectively. This variation in chromosomal length is similar to that found in natural isolates of *E. coli* [43,44] and classifies *L. lactis* among bacterial species with high genome diversity.

Similar results were obtained by Passerini et al. [41] in a Pulsed-Field Gel Electrophoresis (PFGE) study of 36 strains of the subspecies *lactis* isolated from diverse habitats. However, the distribution of chromosome sizes did not correlate with the MLST-based phylogeny. Indeed, marked differences in chromosome size were observed in both the domesticated and the environmental lineages. This suggests that “domestication” does not automatically reduce the genome in this subspecies as a consequence of its adaptation to growth in milk. In contrast, Kelly et al. [45] observed the influence of the origin of the strain on the length of the chromosome. This study comprised 80 strains and the smallest chromosome sizes were found in dairy strains, among which, those of the “true” subspecies *cremoris* (*cremoris* genotype and Cremoris phenotype) had the smallest chromosomes. This reductive chromosome evolution might be a consequence of adaptation of the subspecies *cremoris* to milk environments.

Plasmids contribute significantly to the genome content of *L. lactis* as they can account for 4.7% and 8.4% of the genome within the *lactis* and *cremoris* subspecies respectively. Recent sequence data from NCBI identified 96 completely sequenced lactococcal plasmids. Examination of the plasmid content of 150 dairy starters revealed a mean of seven plasmids per strain [45]. Genomic analysis of the *L. lactis* subsp. *cremoris* UC509.9 strain revealed that extrachromosomal DNA represents more than 200 kb distributed in eight plasmids [46]. In several cases, plasmids have been shown to be transmissible by conjugation, a natural process of DNA transfer that could be exploited in the dairy industry to improve strains by conferring new desirable phenotypes. In contrast, in non-dairy isolates, the average was only two plasmids per strain with larger plasmids (>10 kb) [45]. Plasmid-encoded genes can harbor important technological traits such as proteinase activity, lactose utilization, bacteriophage resistance and bacteriocin production [47,48,49,50,51,52]. Some enhance flavor via citrate utilization. The *citP* gene, which encodes a citrate permease involved in this pathway, is often located on a plasmid [53]. Glutamate dehydrogenase encoding genes are also of interest in the dairy industry. Indeed, this activity stimulates amino acid catabolism in LAB by supplying the 2-oxoglutarate required for amino acid transamination, which is the first step in the conversion of amino acid into aroma compounds. This gene has been observed on plasmid sequences derived from plant and raw milk isolates [54,55]. Natural plasmids harbor food-grade selectable markers, such as copper or cadmium resistance, which are the subject of considerable interest [49,54,56]. Besides these technological properties, plasmids appear to harbor genes that are beneficial for the colonization of specific niches. This is the case for exopolysaccharides (EPS), which play an important role in plant surface attachment and biofilm formation. Furthermore, the capacity to adhere to intestinal epithelial cells and mucins was described in the plant-derived strain TIL448 by Meyrand et al. [57] and Le et al. [58], respectively. A cluster of genes encoding typical genetic biosynthetic machinery for pili formation was found on a plasmid, thereby conferring adhesion and muco-adhesion capacity to the strain. Recently, a novel 12 kb plasmid pSH74 from NCDO 712 was found to contain a new type of pilus gene cluster. Overexpression of the pilus gene cluster led to the formation of appendices on the cell surface [56]. Although plasmid localization for useful properties can be considered as a particular benefit, since transfer via conjugation is possible, it should be kept in mind that this might be also a major disadvantage due to instability and easy loss of plasmids [59].

### 3.3. Functional Diversity

*Lactococcus lactis* is known to display a variety of phenotypes. This phenotypic richness provides a third level of diversity. Generally, there is no correlation between phenotype and genetic lineage and genetically closely related strains do not necessarily share the same phenotypes. For instance, the *lacE* gene, associated with the capacity to consume the lactose, is widely distributed among both domesticated and environmental strains of the subsp. *lactis* [41]. Likewise, diversity in robustness during heat or oxidative stress has been reported in 39 *L. lactis* strains isolated from diverse habitats [60] and does not appear to be related to a specific *lactis* or *cremoris* genotype. This phenotypic robustness is associated with the absence/presence of pattern genes in a collection of strains. For example, the presence of genes encoding a cellobiose transporter (*yidB*), a signal recognition particle receptor protein (*ftsY*) and two hypothetical proteins (*ymgH* and *ymgI*) were associated with the ability to survive oxidative stress. Similarly, the presence/absence of a gene encoding a manganese transporter (*mtsC*) was correlated with resistance of the strains to heat stress. A more exhaustive genomic analysis would have been able to explain this functional diversity, at least to a certain extent. Within the *L. lactis* subsp. *lactis* A12 and KF147 genomes, additional genes encode a range of functions related to the utilization of plant sugars [61,62]. In contrast to industrial dairy starters, these strains efficiently metabolize arabinose and raffinose, and the raffinose-metabolism associated pathway differs in the two strains (cf. Section 4.1).

Besides the presence/absence of genes (related to the accessory genome), inactivation or differential regulation of conserved genes may be the basis of phenotypic diversity. To compare the biochemical properties of 20 strains belonging either to *lactis* or *cremoris* subspecies, Fernandez et al. [39] identified the enzymatic activities of 20 enzymes using the API ZYM and API 20 Strep systems (bioMérieux, Montalieu-Vercieu, France). For all of the strains, a lack of activity was observed in 10 out of the 20 enzymes studied, reflecting either the absence of related genes or their inactivation. For the other enzymes, activity levels for a given enzyme varied considerably among strains and between genotypes. Overall, the level of activity of the *cremoris* genotype was higher than that of the *lactis* genotype. These results could be correlated to a more extensive analysis of strain-specific variations in the activities of the enzymes [63]. Eighty-four *L. lactis* strains from diverse origins were chosen to quantify the specific activity of five enzymes known for their impact on flavor formation (aminopeptidase *N*,X-propyl-dipeptidyl aminopeptidase, branched-chain aminotransferase, hydroxyisocaproic acid dehydrogenase and esterase). Two types of media were used to assess the extent of conservation of the regulatory mechanisms between closely related strains. The authors defined the environment-dependent and strain-specific variations of enzymes activities as “the regulatory phenotype”. This term encompasses the cumulative effects of key mechanisms including transcription, translation or any other allosteric factors that influence enzymatic activity. The data revealed that four out of the five activities measured produced very diverse regulatory responses, clearly showing that regulation differed according to the environmental conditions in a strain-specific manner. This suggests very diverse regulatory characteristics in individual strains and highlights the possibility to reveal various phenotypes. Thus, in a phenotypic screening, the conditions used must be taken into account, and, in addition, conditions used should be as close as possible to those encountered in the process of interest.

The multi-phenotypes expressed by a strain in a specific environment may be a way to differentiate genetically closely related strains. To this end, Dhaisne et al. [64] selected 82 variables as important dairy features, including physiological indicators of the milking process (growth, acidification) and extracellular metabolites, some of which are involved in flavor. These authors tested the variables in nine *L. lactis* subsp. *lactis* strains belonging to the domesticated group with low genetic diversity and the ability to grow in milk. Twenty variables were identified as phenotypic markers that would make it possible to clearly discriminate between strains and to demonstrate their phenotypic uniqueness in this environment. These phenotypic markers were linked to glycolysis, proteolysis and lipolysis, three metabolic pathways involved in flavor production, and highlight the strain-dependent regulation of these pathways.

## 4. From Genome to Phenotype: Original Functions Explained Using an Integrated Approach

### 4.1. Range of Raffinose Metabolism

The original habitat of *L. lactis* is believed to be plants because environmental strains have the capacity to metabolize many plant-derived carbohydrates while the domesticated ones cannot. Several studies have highlighted the ability of environmental strains to use arabinose, xylose, maltose, galacturonate and α-galactosides including melibiose, stachyose and raffinose [39,48]. In the case of raffinose metabolism, genetic features associated with this phenotype are strain dependent. Indeed, the two environmental strains KF147 and A12 metabolize raffinose in two different ways. In the genome of the KF147 strain, a gene cluster for α-galactoside uptake, breakdown and D-galactose conversion via the Leloir pathway has been described: *fbp-galR-aga-galK-galT-purH-agaRCBA-sucP* [65]. Part of this α-galactoside gene cluster, *fbp-galR-aga-galK-galT*, closely resembles (90 to 94% nucleotide identity) that of *Lactococcus raffinolactis* ATCC 43920 [66], a species that naturally degrades raffinose. The cluster is located on a 51 kb transposon, which could be transferred to the MG1363 strain via conjugation, conferring the capacity to use α-galactosides [67]. In contrast, in the A12 strain, genes related to raffinose metabolism differ from those in KF147 [18]. Firstly, these genes are duplicated on the genome, one copy being hosted by a 42 kb plasmid and the other by a 69 kb plasmid. Secondly, α-galactosidase (*aga*) and sucrose phosphorylase (*sucP*) are present but only share, respectively, 52% and 65% nucleotide identity with those of KF147, suggesting an xenologous origin. Thirdly, a putative transporter has been described upstream of these genes. The original structure of the transporter, corresponding to a translational fusion of permease and kinase domains, differs from that of the putative raffinose ABC transporter (encoded by *agaA*, *agaB* and *agaC* in the KF147 strain) and the putative PTS system in the *L. raffinolactis* ATCC 43920.

**Figure 2:**
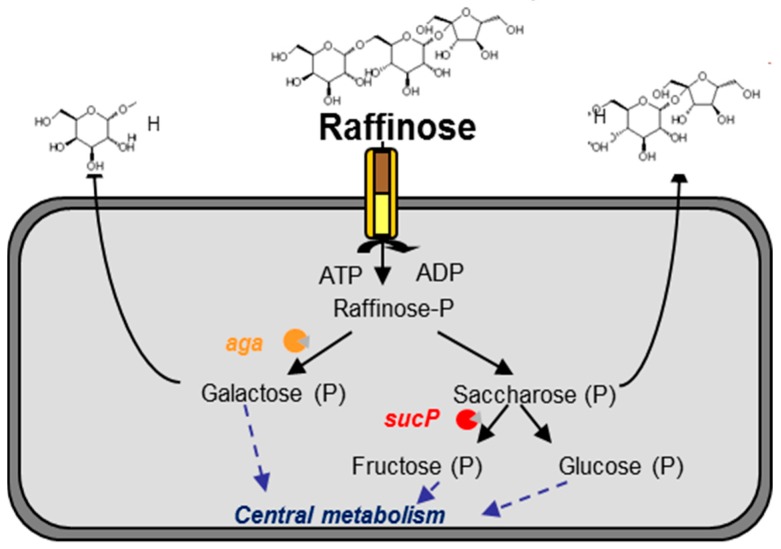
Putative raffinose metabolism in the environmental *L. lactis* subsp. *lactis* A12 strain.

Genomic analysis associated with physiological data allowed the A12 raffinose pathway to be partly elucidated (Figure 2): the transporter is hypothesized to manage both the uptake and the phosphorylation of raffinose. According to this hypothesis, raffinose is cleaved into galactose and saccharose by α-galactosidase. Only 50% of galactose is hypothesized to be consumed by the cell via the Leloir pathway, the remaining 50% being excreted into the medium. Saccharose is more efficiently used by the cell than galactose since only 30% is released into the medium and 70% would be intracellularly cleaved into fructose and glucose by the sucrose phosphorylase. If the physiological data clearly demonstrated the existence of the α-1,6 hydrolysis of raffinose, further investigations are required to propose a potential β-1,2 hydrolysis of raffinose into melibiose and fructose. This particular metabolism would confer a competitive advantage to this strain and enable trophic links with other members of the natural ecosystem.

### 4.2. Different Types of Diacetyl/Acetoin Production

**Figure 3:**
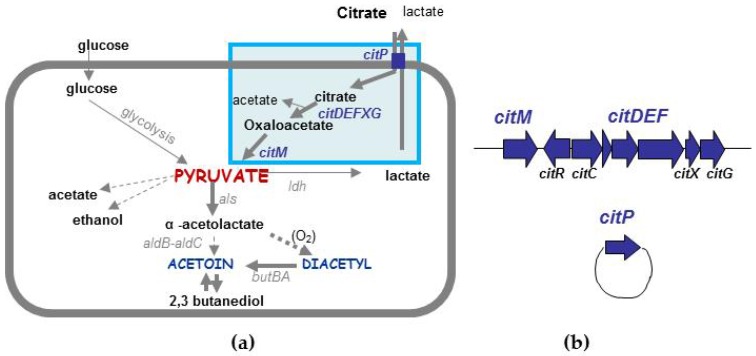
Main contributors to diacetyl/acetoin production. (**a**) pathways involved in citrate metabolism and aroma production. Pyruvate is a key intermediate. (**b**) chromosomal citrate operon and plasmidic *citP* gene involved in citrate transport.

Thanks to their creamy and buttery flavor notes, diacetyl and acetoin are essential components of dairy products. In *L. lactis* subsp. *lactis*, aroma production is associated with the capacity to metabolize citrate, and diacetyl production is proportional to citrate consumption in aerobiosis [68]. The Diacetylactis biovar encompasses strains that have this pathway and this metabolism has been exhaustively described in the dairy strain CRL264 isolated from cheese [69]. Citrate is transported by the plasmid-encoded citrate permease CitP, while genes encoding its intracellular metabolism are located in a large chromosomal cluster (Figure 3b). After its uptake in the cell, citrate is cleaved into acetate and oxaloacetate by the citrate lyase (CitDEF) and its auxiliary proteins (CitC, CitX and CitG). Oxaloacetate is subsequently decarboxylated to pyruvate by the oxaloacetate decarboxylase, CitM. Citrate utilization leads to the accumulation of pyruvate that can be rerouted to two alternative pathways: one generates acetate and/or ethanol and formate, the other generates diacetyl and acetoin. In the second case, two molecules of pyruvate are condensed into α-acetolactate, which is either converted into diacetyl (spontaneous oxydative decarboxylation) or into acetoin (Figure 3a) [70]. In the dairy industry, to rapidly assess the potential of a strain for the production of aroma compounds, citrate utilization is investigated, either by growth on the Kempler and McKay (KMK) medium [71] or by PCR amplification of genes related to the citrate pathway, such as *citP*. Indeed, the citrate plasmid is systematically associated with the presence of the chromosomal cluster [72]. These strains are mostly clustered in the “domesticated” ecotype with low genetic diversity, limiting the diversification of starters. Using an integrated approach, Passerini et al. [72] demonstrated that most of both domesticated and environmental strains can produce diacetyl/acetoin. This expands the extent of the biovar Diacetylactis. Depending on the rate of pyruvate synthesis, the kinetics and the amounts of aroma compounds differ among strains. The presence of the citrate pathway, which actually delineates the Diacetylactis biovar, is related to the rapid accumulation of aroma. In such a case, the name “Citrate” biovar might be more appropriate. Other inefficient-citrate-consuming strains can produce as much aroma but through a slower metabolism. In this case, production depends on their glucose fermenting capacity and pyruvate rerouting towards fermentation end products and is strain-dependent, suggesting different modes of regulation. Thus, only considering genomic features does not fully account for the aromatic potential of a strain. Revealing metabolic differences would be easier by analyzing phenotypes than by analyzing the subtle genomic differences, likely responsible for metabolic heterogeneity.

## 5. Technical and Specific Properties of Environmental Strains for New Applications

The diversity of phenotypes expressed by *L. lactis*, and the technological traits associated with environmental strains could be exploited in dairy fermentation. In addition to acidification, the diversity of metabolic pathways and their end-products such as volatile compounds [23] can be used for the development of new starters with original flavor profiles. From a food safety point of view, many environmental strains produce bacteriocins and bacteriocin-like compounds [73].

Emerging evidence suggests that transient food-borne bacteria play a significant role in host health and gut microbiota, as recently illustrated for *L. lactis* [74]. The authors hypothesized that the ingested strain *L. lactis* CNCM I-1631 could either grow in vivo, adhere to the intestinal wall, or both. To support the second option, the functionality of *L. lactis* cell wall proteins was assessed in vivo using a ∆*srtA* mutant [74]. Consistent with these data, the presence of various gene clusters associated with pili biogenesis, their efficient expression—for instance—in the plant TIL448 strain [57] and the ability to adhere to mucin, also conferred by the joint expression of a mucus-binding protein [58], reinforce lactococcal adhesion as a pivotal factor in transient persistence of *L. lactis* in the gut. Intestinal growth of *L. lactis* may also be a key parameter for increased fitness in the intestine. It requires carbon sources such as mucin-derived carbohydrates and particularly *N*-acetylglucosamine and mannose, which are made available in the gut distal part by commensal mucus degrading bacteria and are then appropriate sugars for exogenously applied bacteria [75]. In mono-associated mice, *L. lactis* was shown to colonize and thrive in the digestive tract, notably through a shift in the gut distal part in lactococcal metabolism from lactose catabolism to *N*-acetylglucosamine and the utilization of mannose [76]. In line with these findings, 151 strains from diverse origins and belonging to the *lactis* and *cremoris* subspecies were screened for their ability to degrade mucin-derived carbohydrates, including fucose, galactose, *N*-acetylglucosamine, *N*-acetylgalactosamine and mannose (vs. lactose and glucose). Interestingly, 10% and 90% of strains were able to metabolize *N*-acetylgalactosamine and galactose, respectively, and none were able to grow on fucose and 100% efficiently degraded *N*-acetylglucosamine and mannose (unpublished data). Moreover, using the same *L. lactis* collection, a wide range of gamma-aminobutyric acid (GABA) production was observed (unpublished data). GABA, a product of glutamate decarboxylation by the glutamic acid decarboxylase, has positive effects on human health such as reducing blood pressure [77,78], psychological stress reducing action [79], and modulating renal function [80]. These phenotypic features associated with technological traits provide a challenging basis for exploiting selected *L. lactis* strains in the development of health-promoting dairy products enriched in GABA.

## 6. Conclusions

The purpose of this review was to highlight the natural diversity of *L. lactis.* If phenotypic differences extensively contribute to this diversity, genetic and genomic variability provide an additional level of diversity that is of primary importance in the development of starters. Indeed, mixing strains with different genotypic characteristics will ensure that the many potentialities encoded by the “pan-genome” of the species are covered. Even though some genetic features are not expressed under specific process conditions, they may be of relevance for other processes or applications. To assess this overall diversity, the use of a multi-scale integrated approach spanning from genotype to phenotype is indispensable.

## Figures and Tables

**Table 1:** Eighty-three available *Lactococcus lactis* genomes. The data was collected from National Center for Biotechnology Information (NCBI); accessed 18 January 2017 (https://www.ncbi.nlm.nih.gov/genome/genomes/156?); N. D.: not determined

Strain	Subspecies	Date	Genome Size (Mb)	Chrom. Size (Mb)	Number of Plasmids	Protein	Isolation Source
IL1403	*lactis*	2001	2.36559	2.36559	0	2277	Cheese starter culture
KF147	*lactis*	2009	2.63565	2.59814	1	2445	Mung bean sprouts
CNCM I-1631	*lactis*	2011	2.51133		N.D.	2403	Fermented milk
CV56	*lactis*	2011	2.51874	2.39946	5	2378	Vaginal flora
IO-1	*lactis*	2012	2.42147	2.42147	0	2229	Water in kitchen sink drain pit
Dephy 1	*lactis*	2013	2.60355		N.D.	2459	N.D.
KLDS 4.0325	*lactis*	2013	2.59549	2.58925	3	2448	Homemade koumiss
LD61	*lactis bv. diacetylactis*	2013	2.59924		N.D.	2490	Starter culture for dairy fermentation
TIFN2	*lactis bv. diacetylactis*	2013	2.50507		N.D.	2296	Cheese starter
TIFN4	*lactis bv. diacetylactis*	2013	2.55039		N.D.	2349	Cheese starter
YF11	*lactis*	2013	2.52731		N.D.	2328	Dairy
511	*lactis*	2014	2.48081		N.D.	2304	N.D.
1AA59	*lactis*	2014	2.57654		N.D.	2406	Artisanal cheese
AI06	*lactis*	2014	2.39809	2.39809	0	2178	Acai pulp
Bpl1	*lactis*	2014	2.3057		N.D.	2092	Wild flies
CECT 4433	*lactis*	2014	2.57915		N.D.	2290	Cheese
GL2	*lactis bv. diacetylactis*	2014	2.33892		N.D.	2135	Dromedary milk
NCDO 2118	*lactis*	2014	2.59226	2.5546	1	2382	Frozen peas
S0	*lactis*	2014	2.4887	2.4887	0	2311	Fresh raw milk
ATCC 19435	*lactis*	2015	2.54729		N.D.	2373	Dairy starter
CRL264	*lactis bv. diacetylactis*	2015	2.57372		N.D.	2446	Cheese
DPC6853	*lactis*	2015	2.50715		N.D.	2116	Corn
E34	*lactis*	2015	2.37566		N.D.	2217	Silage
K231	*lactis*	2015	2.33604		N.D.	2178	White kimchii
K337	*lactis*	2015	2.44552		N.D.	2263	White kimchii
KF134	*lactis*	2015	2.4634		N.D.	2282	Alfalfa and radish sprouts
KF146	*lactis*	2015	2.57452		N.D.	2408	Alfalfa and radish sprouts
KF196	*lactis*	2015	2.44589		N.D.	2282	Japanese kaiwere shoots
KF201	*lactis*	2015	2.37639		N.D.	2222	Sliced mixed vegetables
KF24	*lactis*	2015	2.61922		N.D.	2483	Alfalfa sprouts
KF282	*lactis*	2015	2.65125		N.D.	2471	Mustard and cress
KF67	*lactis*	2015	2.6843		N.D.	2514	Grapefruit juice
KF7	*lactis*	2015	2.36676		N.D.	2209	Alfalfa sprouts
Li-1	*lactis*	2015	2.47593		N.D.	2303	Grass
LMG 7760	*lactis*	2015	2.24545		N.D.	2072	N.D.
LMG 14418	*lactis*	2015	2.41093		N.D.	2275	Bovine milk
LMG 8520	*lactis*	2015	2.43558		N.D.	2060	Leaf hopper
LMG 8526	*lactis*	2015	2.47749		N.D.	2304	Chinese radish seeds
LMG 9446	*lactis*	2015	2.4884		N.D.	2324	Frozen peas
LMG 9447	*lactis*	2015	2.70754		N.D.	2552	Frozen peas
M20	*lactis*	2015	2.67432		N.D.	2535	Soil
ML8	*lactis*	2015	2.52187		N.D.	2373	Dairy starter
N42	*lactis*	2015	2.74392		N.D.	2540	Soil and grass
NCDO895	*lactis*	2015	2.47306		N.D.	2319	Dairy starter
UC317	*lactis*	2015	2.49842		N.D.	2357	Dairy starter
A12	*lactis*	2016	2.73062	2.6039	4	2487	Sourdough
DRA4	*lactis bv. diacetylactis*	2016	2.45755		N.D.	2283	Dairy starter
JCM 7638	*lactis*	2016	2.39386		N.D.	-	N.D
Ll1596	*lactis*	2016	2.39296		N.D.	2237	Teat canal
NBRC 100933	*lactis*	2016	2.54762		N.D.	2406	N.D
RTB018	*lactis*	2016	2.48665		N.D.	2168	Intestinal content of rainbow trout
NBRC 100931	*hordniae*	2016	2.42828		N.D.	2079	Leaf hopper
SK11	*cremoris*	2006	2.59835	2.43859	5	2412	Dairy
MG1363	*cremoris*	2007	2.52948	2.52948	0	2400	Dairy
NZ9000	*cremoris*	2010	2.53029	2.53029	0	2404	Dairy
A76	*cremoris*	2011	2.5771	2.45262	4	2382	Cheese production
UC509.9	*cremoris*	2012	2.45735	2.25043	8	2188	Irish Dairy
KW2	*cremoris*	2013	2.42705	2.42705	0	2223	Fermented corn
TIFN1	*cremoris*	2013	2.67978		N.D.	2285	Cheese starter
TIFN3	*cremoris*	2013	2.72521		N.D.	2291	Cheese starter
TIFN5	*cremoris*	2013	2.54151		N.D.	2232	Cheese starter
TIFN6	*cremoris*	2013	2.59151		N.D.	2334	Cheese starter
TIFN7	*cremoris*	2013	2.63409		N.D.	2505	Cheese starter
A17	*cremoris*	2014	2.67994		N.D.	2367	Taiwan fermented cabbage
GE214	*cremoris*	2014	2.80103		N.D.	2603	Cheese
HP(T)	*cremoris*	2014	2.26951		N.D.	2042	Mixed strain dairy starter culture
DPC6856	*cremoris*	2015	2.86238		N.D.	2606	Bovine rumen
DPC6860	*cremoris*	2015	2.60744		N.D.	2261	Grass
Mast36	*cremoris*	2015	2.60534		N.D.	2414	Milk from a cow with mastitis
AM2	*cremoris*	2016	2.48157		N.D.	2254	Dairy starter
B40	*cremoris*	2016	2.49846		N.D.	2220	Dairy starter
FG2	*cremoris*	2016	2.58614		N.D.	2260	Dairy starter
HP	*cremoris*	2016	2.39396		N.D.	2132	Dairy starter
IBB477	*cremoris*	2016	2.85035	2.64217	5	2653	Raw milk
KW10	*cremoris*	2016	2.36102		N.D.	2177	Kaanga Wai
LMG 6897	*cremoris*	2016	2.3672		N.D.	2101	Cheese starter
N41	*cremoris*	2016	2.61571		N.D.	2410	Soil and grass
NBRC 100676	*cremoris*	2016	2.34409		N.D.	2093	N.D.
NCDO763	*cremoris*	2016	2.48569		N.D.	2331	Dairy starter
P7266	*cremoris*	2016	2.00015		N.D.	1984	Litter on pastures
SK110	*cremoris*	2016	2.46761		N.D.	2241	Dairy starter
V4	*cremoris*	2016	2.54895		N.D.	2344	Raw sheep milk
WG2	*cremoris*	2016	2.54251		N.D.	2306	Cheese
